# Preeclampsia-associated lncRNA FEZF1-AS1 regulates cell proliferation and apoptosis in placental trophoblast cells through the ELAVL1/NOC2L axis

**DOI:** 10.1186/s13008-023-00101-x

**Published:** 2023-10-23

**Authors:** Xudong Zhao, Fengyun Su, Qing Guo, Xiuhong Tao, Huifeng Wang, Hongling Wang, Qinwen Li, Wangmeng Zhang

**Affiliations:** 1 Department of Obstetrics, The Affiliated Taian City Central Hospital of Qingdao University, No.29, Longtan Road, Taian, 271000 Shandong People’s Republic of China; 2 Department of Pharmacy, The Second Affiliated Hospital Of Shandong First Medical University, Taian, 271000 Shandong People’s Republic of China; 3 Intensive Care Unit, The Affiliated Taian City Centeral Hospital of Qingdao University, Taian, 271000 Shandong People’s Republic of China; 4 Department of Ultrasound, The Affiliated Taian City Centeral Hospital of Qingdao University, Taian, 271000 Shandong People’s Republic of China

**Keywords:** Preeclampsia, lncRNA FEZF1-AS1, NOC2L, Trophoblast cells, Apoptosis

## Abstract

**Background:**

LncRNAs have been shown to be involved in and control the biological processes of multiple diseases, including preeclampsia (PE). The impairment of trophoblast cell proliferation is recognized as a significant anomaly contributing to the development of PE. LncRNA FEZF1-AS1 was found downregulated in placental tissues of PE patients. However, the precise regulatory mechanism of FEZF1-AS1 in placental trophoblast proliferation and apoptosis remains unclear.

**Results:**

In this study, we conducted an investigation into the expression levels of FEZF1-AS1 and NOC2L in placental tissues obtained from patients diagnosed with PE. Subsequently, we employed CCK-8 and EdU assays to quantify cell proliferation, while TUNEL staining and western blot for apoptosis-related protein detection to assess apoptosis. Furthermore, the interactions between FEZF1-AS1 and ELAVL1, as well as NOC2L and ELAVL1, were confirmed through the implementation of RIP and RNA pull-down assays. We found a downregulation of lncRNA FEZF1-AS1 and NOC2L in placental tissues of PE patients. Overexpression of FEZF1-AS1 or NOC2L resulted in increased cell proliferation and inhibition of apoptosis, whereas knockdown of FEZF1-AS1 or NOC2L had the opposite effect. In addition, lncRNA FEZF1-AS1 stabilized NOC2L mRNA expression by interacting with ELAVL1. Moreover, partial reversal of the effects of FEZF1-AS1 overexpression on cell proliferation and apoptosis was observed upon suppression of ELAVL1 or NOC2L.

**Conclusions:**

PE related lncRNA FEZF1-AS1 could regulate apoptosis and proliferation of placental trophoblast cells through the ELAVL1/NOC2L axis.

## Highlights

1. LncRNA FEZF1-AS1 and NOC2L modulates the proliferation and apoptosis of trophoblast cells.
2. FEZF1-AS1 stabilizes the mRNA stability of NOC2L by interacting with ELAVL1.
3. FEZF1-AS1/ELAVL1/NOC2L axis regulates trophoblast cell proliferation and apoptosis.

## Background

Preeclampsia (PE), an idiopathic hypertensive syndrome, is a significant contributor to adverse outcomes such as hemorrhage, abortion, preterm birth, fetal, and maternal death, occurring after the 20th week of pregnancy [1]. Surprisingly, the global morbidity of PE is approximately 4%, with perinatal morbidity among pregnant women accounting for half of this total [2]. In the absence of prompt and effective treatment, PE can lead to multiple organ failure reactions, including impairment of liver and kidney function [3]. The occurrence of PE is closely associated with dysfunction of the placental trophoblas. Moreover, the primary pathological mechanisms underlying PE encompass the apoptosis of placental trophoblast cells, incomplete invasion, and abnormal remodeling of the spiral artery within the uterus. The heightened apoptosis of trophoblast cells in the placenta has been widely regarded as the principal etiology of of PE [4]. Simultaneously, numerous studies have suggested that the aberrant expression of multiple genes associated with PE contributes to its development [5, 6]. Therefore, by investigating genes associated with the pathogenesis of PE, a deeper comprehension of the mechanism influencing trophoblast cell apoptosis in PE can be attained, thereby providing a more substantial theoretical foundation and guidance for the management of PE.

Long non-coding RNA (lncRNAs) are a class of RNA molecules whose transcript length exceeds 200nt [7]. LncRNAs regulate gene expression through various mechanisms, including chromatin modification, post-transcriptional modification, and transcriptional regulation [8]. A large body of research indicates that lncRNAs play an important role in PE. For example, researches have shown that lncRNA TCL6, lncRNA-loc391533, and lncRNA CCAT1 promote the progression of preeclampsia by regulating trophoblastic proliferation [9–11]. LncRNA FEZ family zinc finger 1 antisense RNA 1 (FEZF1-AS1) acts as a cancer-promoting factor and plays a crucial role in cancer development [12]. And researches have manifested that the lncRNA FEZF1-AS1 can inhibit cell apoptosis [13, 14]. At the same time, we discovered that lncRNA FEZF1-AS1 exhibited differential expression in the clinical setting (preliminary work) and had not been previously documented in PE. Furthermore, the protective mechanism of lncRNA FEZF1-AS1 in the pathogenesis of PE has not been thoroughly investigated.

NOC2 like nucleolar associated transcriptional repressor (NOC2L) was initially discovered as a a suppressor of in vivo histone acetyltransferase. It functions as a transcriptional co-repressor of p53 and regulates the apoptosis mediated by p53 [15]. The regulation of the p53 pathway affects the apoptosis of trophoblast cells in vitro. Moreover, NOC2L was found downregulated in placental tissues from patient with PE (preliminary work). Generally, lncRNAs have been shown to interact with RNA-binding proteins to regulate downstream mRNA stability [16]. RNA-binding protein ELAV like RNA binding protein 1 (ELAVL1) is also known as HuR [17]. There is a study that showed lncRNA RMST enhances DNMT3 expression by binding with ELAVL1 [18]. Bioinformatics predicts that ELAVL1 has binding sites with lncRNA FEZF1-AS1 and NOC2L. Thus, further study is needed to examine the influence of FEZF1-AS1 on the stability of NOC2L through the recruitment of ELAVL1.

Therefore, we hypothesize that FEZF1-AS1 stabilizes NOC2L by specifically binding to ELAVL1, thereby promoting trophoblast proliferation, inhibiting apoptosis, and alleviating PE disease. In other words, the lncRNA FEZF1-AS1 may alleviate PE disease by regulating trophoblast apoptosis and proliferation.

## Results

### FEZF1-AS1 promotes trophoblast cell proliferation and inhibits apoptosis

**Fig. 1:**
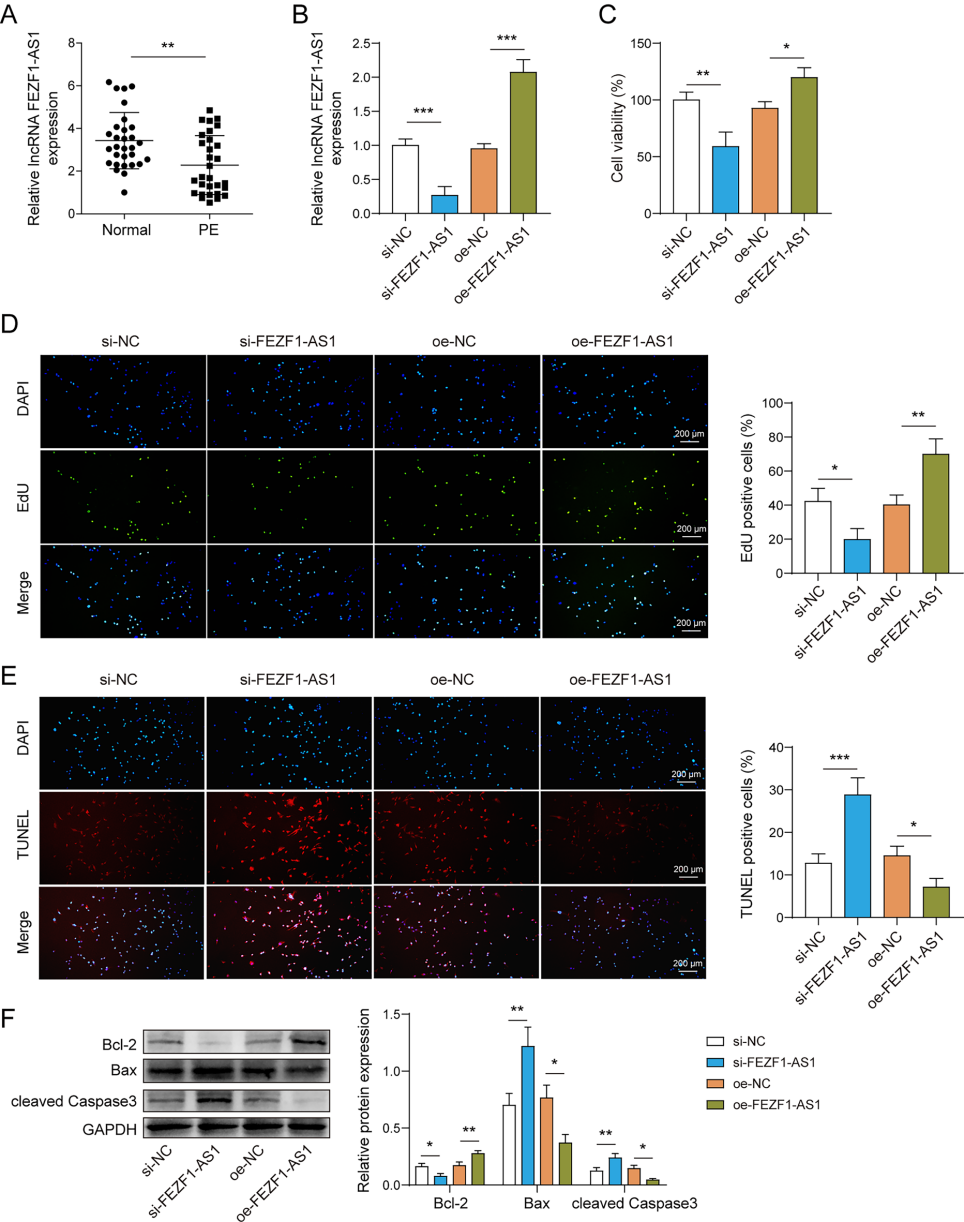
FEZF1-AS1 promotes proliferation and inhibits apoptosis of trophoblast cells. In order to investigate the disparity in the expression of FEZF1-AS1 in PE and normal placental tissues, the following experiment was conducted. **A** The expression of FEZF1-AS1 was detected by qPCR, n = 30, **p < 0.01. FEZF1-AS1 was knocked down or overexpressed in HTR-8/SVneo cell lines, and they were grouped as follows: si-NC, si-FEZF1-AS1, oe-NC, and oe-FEZF1-AS1. **B** The silencing and overexpression efficiency of FEZF1-AS1 was evaluated using qPCR. **C** The CCK-8 assay was used to measure cell viability. **D** The EdU assay was used to detect cell proliferation. **E** Apoptosis was detected using the TUNEL assay. **F** The protein expressions of Bcl-2, Bax, and cleaved Caspase3 were detected by western blot. n = 3, *p < 0.05, **p < 0.01, ***p < 0.001

We used qPCR to analyze the expression of FEZF1-AS1 in PE and control (normal placenta) tissues. As shown in Fig. 1A, the expression of FEZF1-AS1 in the PE group was significantly decreased. In order to investigate the effects of FEZF1-AS1 on trophoblast cell proliferation and apoptosis, si-NC, si-FEZF1-AS1, oe-NC, and oe-FEZF1-AS1 were transfected into HTR-8/SVneo cells. The qPCR results indicated that si-FEZF1-AS1 and oe-FEZF1-AS1 were effectively transfected (Fig. 1B). In terms of cell phenotype, cell viability and proliferation were measured using the CCK-8 assay and EdU assay. The results showed that si-FEZF1-AS1 reduced cell viability and proliferation, while oe-FEZF1-AS1 accelerated cell viability and proliferation (Fig. 1C, D). Inversely, the TUNEL assay results indicated that si-FEZF1-AS1 significantly promoted apoptosis, while oe-FEZF1-AS1 showed the opposite trend (Fig. 1E). Simultaneously, si-FEZF1-AS1 down-regulated Bcl-2 protein level, up-regulated Bax and cleaved Caspase3, oe-FEZF1-AS1 resulted in an opposite protein expression pattern (Fig. 1F). Taken together, the lncRNA FEZF1-AS1 accelerated HTR-8/SVneo cell proliferation and inhibited apoptosis.

## NOC2L promotes the proliferation and inhibits the apoptosis of trophoblast cells

**Fig. 2:**
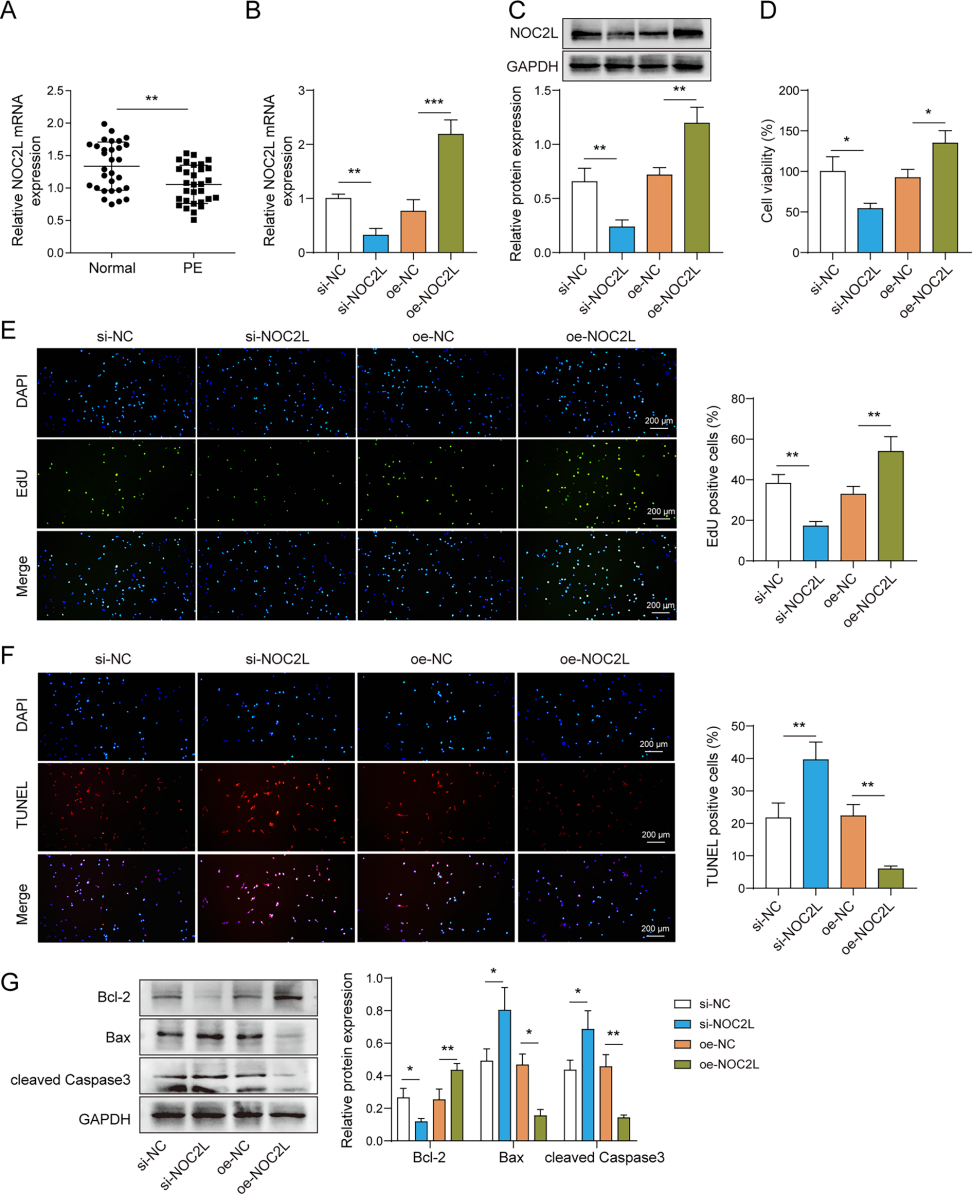
NOC2L promotes the proliferation and inhibits the apoptosis of trophoblast cells. For investigating the differential expression of NOC2L in PE and normal placental tissues, the following experiment was conducted. **A** The expression of NOC2L was detected using qPCR, n = 30, **p < 0.01. NOC2L was knocked down or overexpressed in HTR-8/SVneo cell lines, which were grouped as follows: si-NC, si-NOC2L, oe-NC, and oe-NOC2L. **B** The silencing and overexpression efficiency of NOC2L was evaluated using qPCR. **C** The silencing and overexpression efficiency of NOC2L was verified using western blot. **D** The CCK-8 assay was used to measure cell viability. **E** The EdU assay was used to detect cell proliferation. **F** Apoptosis was detected using the TUNEL assay. **G** The protein expressions of Bcl-2, Bax, and cleaved caspase-3 were detected by western blot. n = 3, *p < 0.05, **p < 0.01, ***p < 0.001

We performed qPCR on PE and normal samples, and identified that NOC2L was significantly downregulated in PE tissues (Fig. 2A). This implies that NOC2L may play a significant role in PE diseases. Therefore, the overexpression and knockdown treatment of NOC2L in HTR-8/SVneo cells are classified as follows: si-NC, si-NOC2L, oe-NC, and oe-NOC2L. Firstly, the qPCR and western blot results indicated overexpression of NOC2L promoted the expression levels of NOC2L mRNA and protein, while silencing of NOC2L inhibited the expression levels (Fig. 2B, C). Then, si-NOC2L significantly restrained cell viability and cell proliferation but facilitated apoptosis, while oe-NOC2L showed the opposite trend (Fig. 2D–F). Moreover, si-NOC2L downregulated the protein level of Bcl-2, but upregulated Bax and cleaved Caspase3, while oe-NOC2L reversed this trend (Fig. 2G). Therefore, NOC2L could promote the proliferation of trophoblast cells and inhibit apoptosis.

### FEZF1-AS1 and NOC2L bind to ELAVL1

**Fig. 3:**
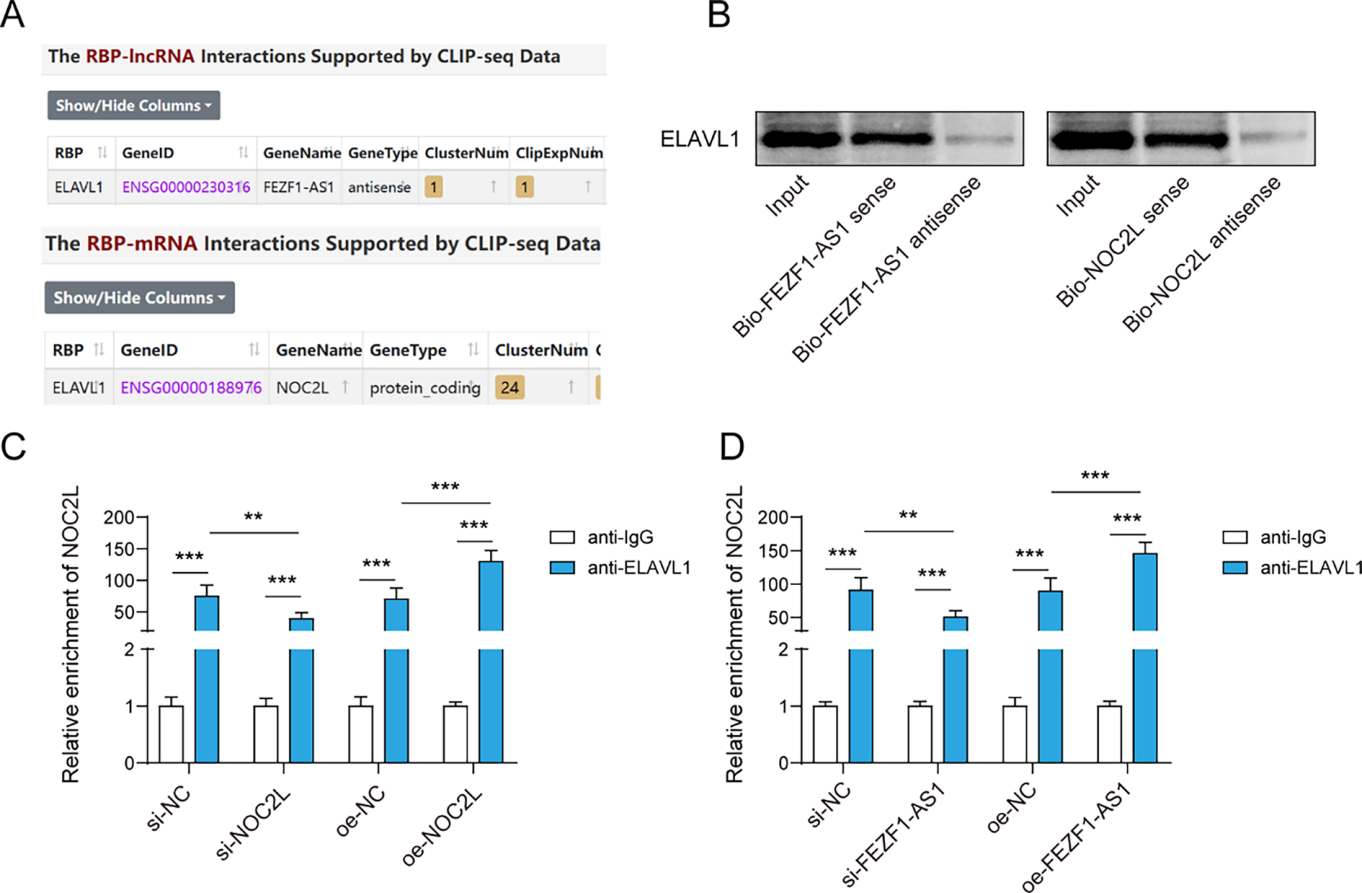
FEZF1-AS1 and NOC2L bind to ELAVL1. **A** ENCORI predicted that FEZF1-AS1 and NOC2L have binding sites with ELAVL1. In order to verify the interaction between FEZF1-AS1 and NOC2L with ELAVL1, the following experiment was conducted. **B** RNA pull-down verified the binding relationship between ELAVL1 and FEZF1-AS1, as well as ELAVL1 and NOC2L. NOC2L was knocked down or overexpressed in HTR-8/SVneo cell lines, which were grouped as follows: si-NC, si-NOC2L, oe-NC, and oe-NOC2L. **C** The RIP assay was used to detect the effect of NOC2L on the interaction between ELAVL1 and NOC2L. FEZF1-AS1 was knocked down or overexpressed in HTR-8/SVneo cell lines, and they were grouped as follows: si-NC, si-FEZF1-AS1, oe-NC, oe-FEZF1-AS1. **D** The RIP assay was used to detect the effect of FEZF1-AS1 on the interaction between ELAVL1 and NOC2L. n = 3, **p < 0.01, ***p < 0.001

FEZF1-AS1 and NOC2L have binding sites with ELAVL1, which predicted by ENCORI (http://starbase.sysu.edu.cn/) (Fig. 3A). Besides, to unveil the combination of FEZF1-AS1, NOC2L, and ELAVL1, RNA pull-down assay verified that the enrichment of ELAVL1 in Bio-FEZF1-AS1 sense and Bio-NOC2L sense groups, indicating that ELAVL1 could bind to FEZF1-AS1 or NOC2L (Fig. 3B). To further confirm the binding of FEZF1-AS1 and NOC2L to ELAVL1, we detected the interaction between ELAVL1 and NOC2L through knockdown and overexpression of NOC2L in the RIP experiment. Silencing NOC2L inhibited the interaction between NOC2L and ELAVL1, but oe-NOC2L increased the content of ElAVL1-NOC2L complex (Fig. 3C). Furthermore, si-FEZF1-AS1 significantly inhibited the interaction between ELAVL1 and NOC2L, while overexpression of FEZF1-AS1 promoted their interaction (Fig. 3D). In summary, FEZF1-AS1 interacted with ELAVL1 and affected the interaction between ELAVL1 and NOC2L.

### The interaction between FEZF1-AS1 and ELAVL1 increases the stability of NOC2L mRNA

**Fig. 4:**
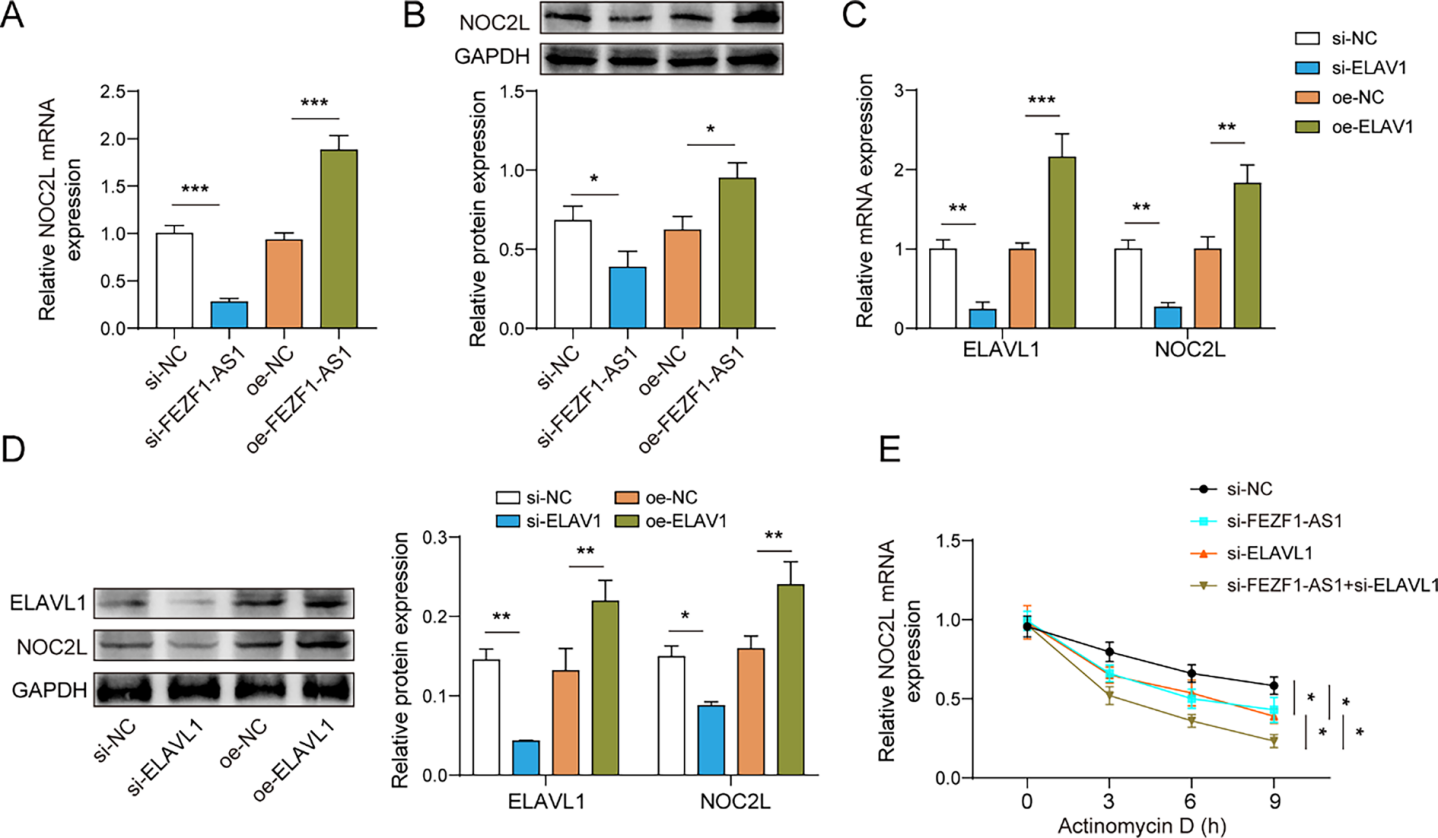
The interaction between FEZF1-AS1 and ELAVL1 increased the stability of NOC2L mRNA. FEZF1-AS1 was knocked down or overexpressed in HTR-8/SVneo cell lines, grouped as follows: si-NC, si-FEZF1-AS1, oe-NC, and oe-FEZF1-AS1. **A, B** The mRNA and protein expression levels of NOC2L were detected by qPCR and western blot techniques. ELAVL1 was knocked down or overexpressed in HTR-8/SVneo cell lines, grouped as follows: si-NC, si-ELAVL1, oe-NC, and oe-ELAVL1. **C, D** The mRNA and protein expression of NOC2L and ELAVL1 were detected by qPCR and western blot assays. FEZF1-AS1 and/or ELAVL1 were knocked down in HTR-8/SVneo cell lines and grouped as follows: si-NC, si-FEZF1-AS1, si-ELAVL1, and si-FEZF1-AS1 + si-ELAVL1. **E** The effects of FEZF1-AS1 and ELAVL1 on the stability of NOC2L mRNA were detected by qPCR. n = 3, *p < 0.05, **p < 0.01, ***p < 0.001

In order to further understand the regulatory effect of the combination of FEZF1-AS1 and ELAVL1 on NOC2L, knockdown and overexpression of FEZF1-AS1 were performed in HTR-8/SVneo cells. The knockdown of FEZF1-AS1 inhibited the mRNA and protein expression levels of NOC2L, whereas the overexpression of FEZF1-AS1 promoted the mRNA and protein expression levels of NOC2L (Fig. 4A, B). Additionally, si-NC, si-ELAVL1, oe-NC, and oe-ELAVL1 were transfected into HTR-8/SVneo cell lines, si-ELAVL1 downregulated the expression levels of ELAVL1 and NOC2L, while oe-ELAVL1 played the opposite role (Fig. 4C, D). Then, si-FEZF1-AS1 or si-ELAVL1 accelerated the decay of NOC2L mRNA after the addition of actinomycin D. Moreover, the degree of NOC2L mRNA decay greatly increased under the combined action of si-FEZF1-AS1 and si-ELAVL1 (Fig. 4E). To sum up, FEZF1-AS1 enhanced the stability of NOC2L mRNA by recruiting ELAVL1.

### The signaling axis of FEZF1-AS1/ELAVL1/NOC2L influenced trophoblast cell proliferation and apoptosis

**Fig. 5:**
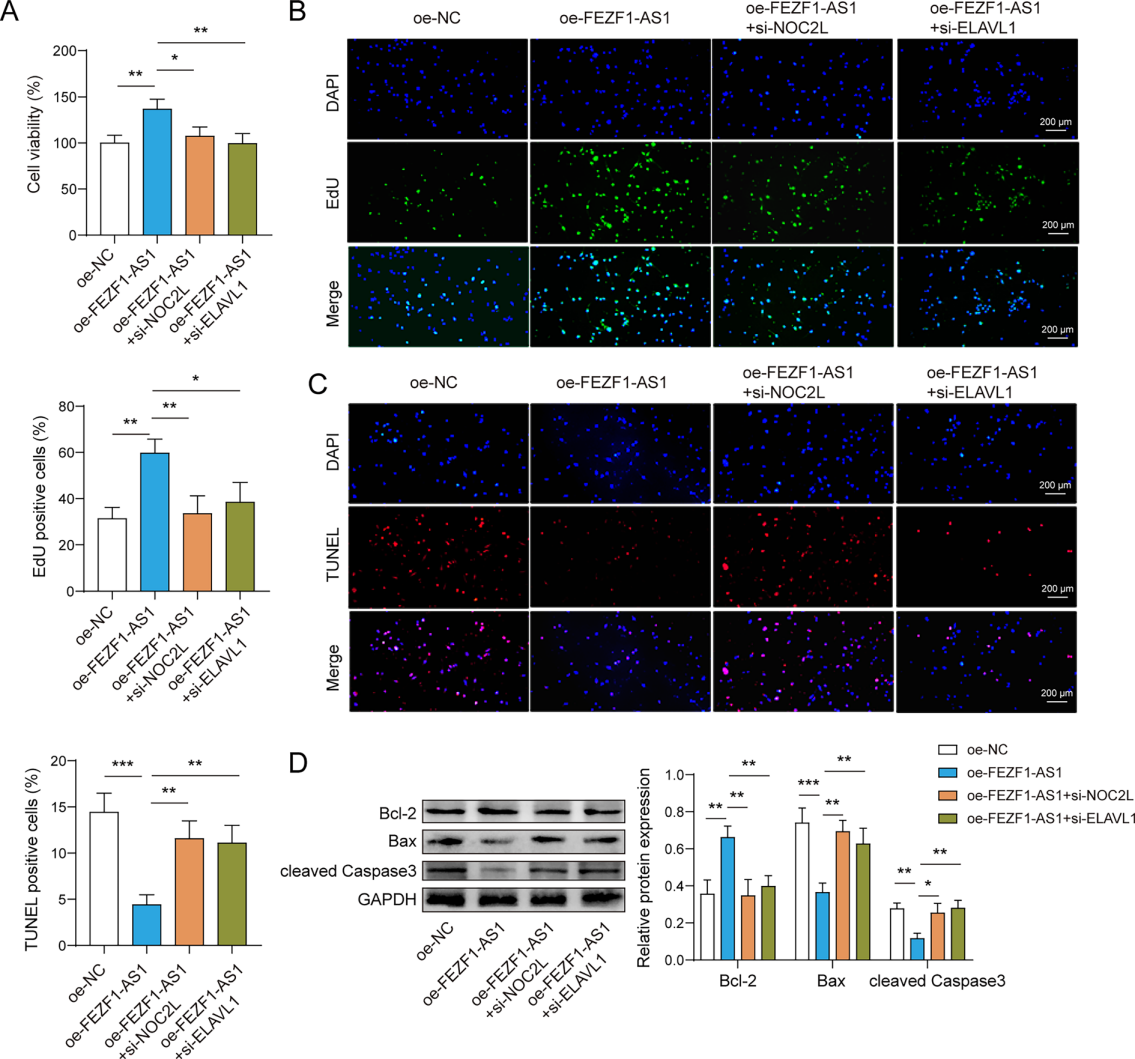
The FEZF1-AS1/ELAVL1/NOC2L signaling axis influences the proliferation and apoptosis of trophoblast cells. To investigate the regulation of trophoblast cell proliferation and apoptosis by the FEZF1-AS1/ELAVL1/NOC2L signaling axis, we conducted a recovery experiment. The groups were categorized as follows: oe-NC, oe-FEZF1-AS1, oe-FEZF1-AS1 + si-ELAVL1, and oe-FEZF1-AS1 + si-NOC2L. **A** The CCK-8 assay was used to measure cell viability. **B** The EdU assay was used to detect cell proliferation. **C** Apoptosis was detected using the TUNEL assay. **D** The protein expressions of Bcl-2, Bax, and cleaved caspase-3 were detected by western blot. n = 3, *p < 0.05, **p < 0.01, ***p < 0.001

To investigate the signaling axis of FEZF1-AS1/ELAVL1/NOC2L in trophoblast cells, we transfected oe-NC, oe-FEZF1-AS1, oe-FEZF1-AS1 + si-ELAVL1, and oe-FEZF1-AS1 + si-NOC2L into HTR-8/SVneo cells. Overexpression of FEZF1-AS1 upregulated cell activity and proliferation, and inhibition of NOC2L or ELAVL1 partially reversed these changes (Fig. 5A, B). Overexpression of FEZF1-AS1 inhibited apoptosis, and inhibition of NOC2L or ELAVL1 can partially eliminate the impacts of FEZF1-AS1 on HTR-8/SVneo cell apoptosis (Fig. 5C). Inhibition of NOC2L or ELAVL1 partially reversed the promotion of Bcl-2 and the inhibition of Bax and cleaved Caspase3 expression by overexpression of FEZF1-AS1 (Fig. 5D). Thus, FEZF1-AS1 facilitated trophoblast cell proliferation and inhibited apoptosis through the ELAVL1/NOC2L signaling axis.

## Discussion

The harms of PE disease include inducing fetal death, fetal dysplasia, and damage to various maternal organs [19]. Recently, a great deal of research has continued to investigate PE, but the specific reasons have not been clarified. Therefore, we have verified the specific molecular mechanism of lncRNA FEZF1-AS1 in PE. It has been found that lncRNA FEZF1-AS1 regulates the expression of NOC2L by interacting with ELAVL1. This interaction promotes trophoblast cell proliferation and inhibits apoptosis.

More and more evidence manifested that lncRNAs developed a significant role in PE [20]. Different lncRNAs have varying effects on PE by regulating placental tissue vascular repair, trophoblast cell proliferation, and inflammatory factors. Examples of lncRNAs include TUG1 [21], UCA1 [22], and uc003fir [23]. In previous studies, it has been shown that FEZF1-AS1 is involved in female diseases, specifically affecting cervical cancer cell proliferation [24–26]. However, it is yet to be determined whether it can also affect trophoblast cells. Our results revealed that the expression of FEZF1-AS1 in PE tissues was lower than that in normal uterine tissues. Additionally, trophoblast cell proliferation was promoted, and the apoptosis of trophoblast cells was inhibited after overexpressing FEZF1-AS1. FEZF1-AS1 was involved in trophoblast proliferation and apoptosis, which is consistent with the biological role of FEZF1-AS1 in tumor progression.

NOC2L, also known as NIR, is involved in cell proliferation and apoptosis [15]. One possible explanation for its effect on cell proliferation is that NOC2L acts as a suppressor of p53, thereby reversing the detrimental impact of p53 overexpression on embryonic development [27]. Our results also confirmed that overexpression of NOC2L could enhance cell proliferation and reduce apoptosis in trophoblasts. It is well known that lncRNAs are involved in regulating the expression of target genes by interacting with RNA-binding proteins [28]. In the present experiment, ELAVL1 was selected and confirmed as a potential RNA-binding protein for FEZF1-AS1 and NOC2L. Moreover, extensive evidence suggests that ELAVL1, also known as HuR, is an RNA-binding protein that stabilizes mRNA and prevents gene degradation by binding to conserved AU-rich elements (AREs) within 3’UTRs [29]. It has been shown that lncRNA HMS recruits ELAVL1 to stabilize the 3'-UTR of HOXC10 mRNA [30]. In our findings, knockdown of ELAVL1 reduced the expression of NOC2L at both the RNA and protein levels. Using RIP and RNA pull-down experiments, combined with the examination of the effect of FEZF1-AS1 upregulation on NOC2L expression, we have concluded that FEZF1-AS1 enhances NOC2L mRNA stability by binding with ELAVL1. And FEZF1-AS1 regulates trophoblast apoptosis through the ELAVL1/NOC2L axis.

In short, this study demonstrated that lncRNA FEZF1-AS1 is lowly expressed in PE patients, and FEZF1-AS1 acts as a protective factor by promoting trophoblast cell proliferation and inhibiting apoptosis. It promotes the upregulation of NOC2L expression by binding to ELAVL1. Thus, the FEZF1-AS1/ELAVL1/NOC2L axis may be a valuable target for therapy in patients with PE. Unfortunately, this study has not been further validated through animal experiments, which will be the focus of our future research. Additionally, the sample size would be increased, and including more patient characteristic information.

## Conclusions

**Fig. 6:**
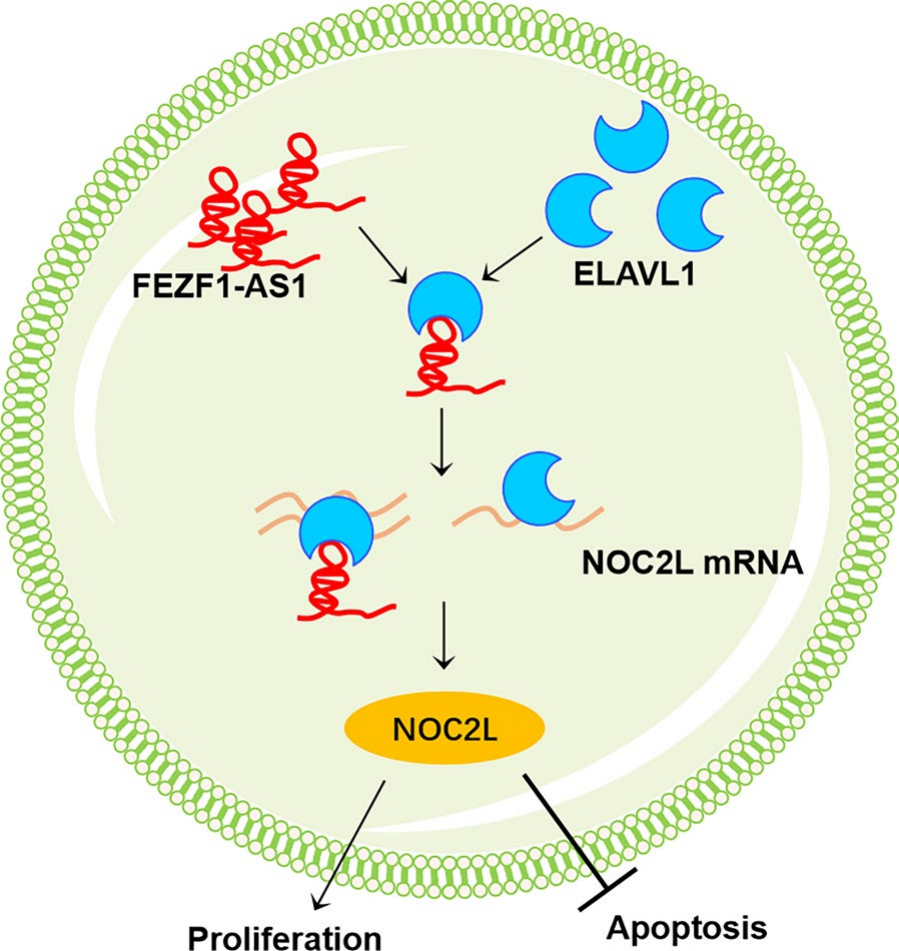
Schematic representation of the study. Briefly, lncRNA FEZF1-AS1 enhanced the stability of the mRNA of NOC2L, leading to an increase in its expression. This is achieved through the interaction between FEZF1-AS1 and ELAVL1. As a result, apoptosis was inhibited and proliferation was promoted in trophoblast cells

LncRNA FEZF1-AS1 played a vital role in inhibiting the development of PE disease. Its possible underlying mechanism involves promoting cell proliferation and suppressing apoptosis by bolstering the stability of NOC2L mRNA through the recruitment of ELAVL1 (Fig. 6). Our current study suggests that LncRNA FEZF1-AS1 may be considered as an effective target for the treatment of PE. Meanwhile, this study does have a few limitations. Future studies will investigate the effects of overexpressing FEZF1-AS1 or overexpressing NOC2L on a rat model of preeclampsia and observe the levels of placental oxidative stress and vascular endothelial function in the model. Furthermore, future studies need to explore the role of FEZF1-AS1 in the pathogenesis of PE.

## Methods

### Samples collection

The placental tissue was collected from patients with PE and from normal controls (n = 30). This research was authorized by the Medical Ethics Committee of The Affiliated Taian City Central Hospital of Qingdao University (No.202118) with the written informed consent of each participant. Inclusion criteria for the PE group were as follows: (1) diastolic blood pressure ≥ 90 mmHg from two recordings or systolic blood pressure ≥ 140 mmHg; (2) a 24 h urinary protein of ≥ 0.3 g, or a random urinary protein of 1 + or more. Within 5 min of surgical removal of placental tissue, the placenta was cut into 1 cm × 1 cm × 1 cm cubes. The cubes were then washed repeatedly with phosphate buffered saline (PBS) until all blood was removed. The sterile placenta cubes were preserved at − 80 °C for subsequent experiments.

### Human chorionic trophoblast cell culture

HTR-8/SVneo cells (ATCC) were cultured in RPMI-1640 medium (Gibco, USA) supplemented with 10% FBS (Gibco), 100 U/mL penicillin, and 100 μg/mL streptomycin. All cell lines were cultured in a humidified air environment at 37 °C with 5% CO_2_. Small interfering RNAs (si-FEZF1-AS1: CCC ACG AAG UUU AAA GCA UTT, si-NOC2L: GAC CUG AAC UUC CCA GAG A, and si-ELAVL1: AAG AGG CAA UUA CCA GUU UCA), overexpressed vectors (pcDNA3.1-FEZF1-AS1: oe-FEZF1-AS1, pcDNA3.1-NOC2L: oe-NOC2L, and pcDNA3.1-ELAVL1: oe-ELAVL1), and relative negative controls (si-NC: UUC UCC GAA CGU GUC ACG UTT; pcDNA3.1 vector: oe-NC) were obtained from GenePharma (Shanghai, China). According to the manufacturer’s guidelines, cells were transfected with si-RNAs or vectors for 48 h using Lipofectamine 3000 (Invitrogen, USA).

### Cell counting kit-8 (CCK-8) assay

In brief, the medium was replaced with pre-warmed medium (100 µL) per well in 96-well plates. CCK-8 reagents (10 µL; Sigma, USA) were added directly, and then the cells were incubated for 2 h at 37 °C. The absorbance at 450 nm was recorded using a microplate reader (BioTek, Winooski, VT, USA). The untreated cells were normalized in order to calculate the relative cell viability.

### Quantitative real-time polymerase chain reaction (qPCR) assay

The TRIzol (Invitrogen) method was used to extract total RNA from HTR-8/SVneo cells. The PrimeScript RT Reagent Kit with gDNA Eraser (TaKaRa, Japan) was used to reverse transcribe RNA into cDNA. The qPCR assay was performed using the SYBR Green kit (Thermo Fisher Scientific) to measure the amplification of the target genes. The primer sequences were as follows: FEZF1-AS1 (forward: TTA GGA GGC TTG TTC TGT GT; reverse: GCG CAG GTA CTT AAG AAA GA), NOC2L (forward: CGG CAC AAG AAG GAC ACT TTC C; reverse: TCC GTC AAG GTC CAC TGC ATG A), ELAVL1 (forward: TGT TCT CTC GGT TTG GGC GGA T; reverse: TCT TCT GCC TCC GAC CGT TTG T), GAPDH (forward: GTC TCC TCT GAC TTC AAC AGC G; reverse: ACC ACC CTG TTG CTG TAG CCA A). Data analysis was conducted using the 2^−∆∆Ct^ method, with GAPDH serving as the internal reference.

### Western blot assay

The extracted protein samples were transferred onto polyvinylidene difluoride membranes after being loaded onto a sodium dodecyl sulfate/polyacrylamide gel for electrophoresis. The membranes were blocked with 5% bovine serum albumin for 1 h before being incubated with primary antibodies overnight at 4 °C: Bcl-2 (ab182858, 1:2000, Abcam, USA), Bax (ab32503, 1:2000, Abcam), cleaved Caspase3 (ab32042, 1:500, Abcam), ELAVL1 (ab200342, 1:1000, Abcam), NOC2L (ab198171, 1:500, Abcam), and GAPDH (ab181602, 1:10,000, Abcam). The secondary antibody conjugated with horseradish peroxidase was used to incubate the membranes for 1 h. The enhanced chemiluminescence substrate (Bio-Rad, USA) was finally used to detect the protein bands.

### 5-ethynyl-2′-deoxyuridine (EdU) assay

A EdU kit (RiboBio, China) was used to measure cell proliferation following standard procedures. In brief, the treated cells were uniformly seeded in 96-well plates and incubated in a constant temperature environment for 24 h to achieve 60% fusion. 200 µL of a 50 µM EdU solution was added to each well and incubated for 2 h before being discarded. After washing with PBS buffer, the cells were fixed by adding 4% paraformaldehyde for 30 min. The formaldehyde was neutralized by adding a 2 mg/mL glycine solution, and the cells were washed with a PBS buffer. Under dark conditions, 100 µL of Apollo staining reaction solution was added to each well and incubated for 30 min at room temperature. Then, the cells were permeated by adding an osmotic agent (PBS containing 0.5% Triton X-100) and subsequently incubated with DAPI (Invitrogen) to stain the nucleus. The images were captured and analyzed using a fluorescence microscope, and the percentage of EdU-positive cells was calculated.

### TUNEL assay

The trophoblast cells were washed twice with PBS, then fixed with 4% paraformaldehyde. After fixation, the cells were infiltrated with a balancing buffer containing 0.2% Triton X-100. Then, cell DNA fragmentation was measured using the TUNEL method (Beyotime, Shanghai, China), following the manufacturer's manual. Images were obtained using a fluorescence microscope, and the percentage of TUNEL-positive cells was calculated to assess apoptosis.

### RNA pull-down assay

The biotinylated FEZF1-AS1/NOC2L RNA (Bio-FEZF1-AS1-sense/antisense or NOC2L-sense/antisense) was designed and synthesized by Thermo Fisher Scientific (USA). The probes were transfected into HTR-8/SVneo cells for 48 h, then treated with streptavidin magnetic beads (Invitrogen). After washing off the unbound proteins, the RNA–protein complex is eluted from the beads using an eluent. Finally, ELAVL1 enrichment was detected by western blot.

### RNA immunoprecipitation (RIP) assay

For RIP assay, the EZ-Magna RIP Kit was bought from Millipore (Billerica, MA, USA) and utilized in line with the user guide to investigate the interaction between ELAVL1 and NOC2L. Cells were lysed in the polysome lysis buffer and incubated with ELAVL1 antibody or isotype‐matched control antibody (IgG; Millipore) conjugated magnetic beads. The beads were collected and washed, followed by RNA purification from the RNA–protein complex. The co-precipitated RNA enrichment was detected by qPCR and 3 biological replicates were carried out.

### Actinomycin D assay

After transfection, cells were treated with actinomycin D (5 μg/mL) for 0, 3, and 6 h, respectively. Then, cells were collected for total RNA extraction. cDNA was synthesized through reverse transcription, and the levels of target RNA and reference RNA were detected using qPCR.

## Statistical analysis

All data were obtained from at least three replicate experiments and summarized as means ± standard deviation (SD). After evaluating the normal distribution with the Shapiro–Wilk test, statistical analysis was performed using GraphPad Prism 8.0 (GraphPad Software) with either one-way ANOVA for multiple groups or Student's t-test for two groups. p < 0.05 was considered statistically significant.

## Data Availability

The datasets used or analyzed during the current study are available from the corresponding author on reasonable request.

## Abbreviations

PE: Preeclampsia
qPCR: Quantitative real-time polymerase chain reaction
CCK-8: Cell counting kit-8
RIP: RNA Immunoprecipitation
LncRNA: Long non-coding RNA
FEZF1-AS1: FEZ family zinc finger 1 antisense RNA 1
ELAVL1: ELAV like RNA binding protein 1
NOC2L: NOC2 like nucleolar associated transcriptional repressor
EdU: 5-Ethynyl-2′-deoxyuridine
